# Insecticide use pattern and phenotypic susceptibility of *Anopheles gambiae* sensu lato to commonly used insecticides in Lower Moshi, northern Tanzania

**DOI:** 10.1186/s13104-017-2793-4

**Published:** 2017-09-06

**Authors:** Elinas J. Nnko, Charles Kihamia, Filemoni Tenu, Zul Premji, Eliningaya J. Kweka

**Affiliations:** 1 Department of Parasitology and Medical Entomology, Muhimbili University of Health and Allied Sciences, P.O. Box 65011, Dar es Salaam, Tanzania; 2 National Institute for Medical Research, Amani Medical Research Centre, Muheza, P.O. Box 81, Tanga, Tanzania; 3 Tropical Pesticides Research Institute, Division of Livestock and Human Health Disease Vector Control, Mosquito Section, P.O. Box 3024, Arusha, Tanzania; 4 Department of Medical Parasitology and Entomology, School of Medicine, Catholic University of Health and Allied Sciences, P.O. Box 1464 Mwanza, Tanzania

**Keywords:** Resistance ratio, Mortality, Resistance, Insecticides, *Anopheles gambiae* s.l., Tanzania

## Abstract

**Background:**

Evidence of insecticide resistance has been documented in different malaria endemic areas. Surveillance studies to allow prompt investigation of associated factors to enable effective insecticide resistance management are needed. The objective of this study was to assess insecticide use pattern and phenotypic susceptibility level of *Anopheles gambiae* sensu lato to insecticides commonly used in malaria control in Moshi, northern Tanzania.

**Methods:**

A cross-sectional survey was conducted to assess insecticide usage pattern. Data was collected was through closed and open ended questionnaires The WHO diagnostic standard kit with doses of 0.1% bendiocarb, 0.05% deltamethrin, 0.75% permethrin and 4% DDT were used to detect knockdown time, mortality and resistance ratio of wild *A. gambiae* sensu lato. The questionnaire survey data was analyzed using descriptive statistics and one-way analysis of variance while susceptibility data was analysed by logistic regression with probit analysis using SPSS program. The WHO criteria was used to evaluate the resistance status of the tested mosquito populations.

**Results:**

A large proportion of respondents (80.8%) reported to have used insecticide mainly for farming purposes (77.3%). Moreover, 93.3% of household reported usage of long lasting insecticidal nets. The frequently used class of insecticide was organophosphate with chloropyrifos as the main active ingredients and dursban was the brand constantly reported. Very few respondents (24.1%) applied integrated vector control approaches of and this significantly associated with level of knowledge of insecticide use (*P* < 0.001). Overall knockdown time for *A. gambiae* s.l was highest in DDT, followed by Pyrethroids (Permethrin and deltamethrin) and lowest in bendiocarb. *Anopheles gambiae* s.l showed susceptibility to bendiocarb, increased tolerance to permethrin and resistant to deltamethrin. The most effective insecticide against the population from tested was bendiocarb, with a resistance ratio ranging between 0.93–2.81.

**Conclusion:**

Education on integrated vector management should be instituted and a policy change on insecticide of choice for malaria vector control from pyrethroids to carbamates (bendiocarb) is recommended. Furthermore, studies to detect cross resistance between pyrethroids and organophosphates should be carried out.

**Electronic supplementary material:**

The online version of this article (doi:10.1186/s13104-017-2793-4) contains supplementary material, which is available to authorized users.

## Background

In Sub Saharan Africa, species from the *Anopheles gambiae* complex and *Anopheles funestus* groups are the important malaria vectors [1, 2]. Out of the eight members of the *A. gambiae* complex sibling species, *A. gambiae* s.s and *A. arabiensis* are the main malaria vectors across sub-Saharan Africa including Tanzania [1–6]. Malaria is still a major cause of mortality and morbidity in sub-Saharan Africa including Tanzania [7–9]. The government of Tanzania has extensively provided and is scaling up free distribution of long lasting insecticidal nets [10, 11], free anti-malarial [12] and rapid diagnostic kits [13] in all health facilities across the country as a strategy for strengthening malaria control. Vector control constitute a major component of the global strategy for malaria control [14, 15]. Use of long lasting insecticide treated nets (LLINs), indoor residual spraying (IRS) and larviciding are the pillars of malaria vector control programmes [16–19].

Development of Insecticide resistance in targeted vector populations pose a major threat in malaria vector control as it weakens efficiency of insecticide based intervention tools [20–24]. Resistance has been documented in all classes of insecticides used in public health, veterinary and agricultural pests control including pyrethroids, carbamates, organophosphates and organochlorines, [20, 21, 25–27]. Commonly used pesticides in agriculture and public health are organophosphates (such as fenitrothion, malathion and pirimiphos-methyl), organochlorines such as dichlorodiphenyltrichloroethane (DDT), carbamates (such as bendiocarb and propoxur) and pyrethroids (alphacypermethrin, bifenthrin, cyfluthrin, deltamethrin, lambdacyhalothrin, etofenprox). Currently, pyrethroids is the only recommended insecticide class for application in LLINs [28]. Some of these pesticides residues have been found in soil and water from different areas practicing intensive agriculture and pesticides usage for higher yield productivity in vegetable gardens, cotton farms, horticulture and rice field [29, 30]. Cross resistance has been reported between DDT and pyrethroids that weakening the control efforts [30].

Four different insecticide resistance mechanisms have been reported in malaria vector and include; target site resistance, metabolic resistance, behavioral resistance and cuticular resistance [31–34]. Target site and metabolic resistance mechanisms are the most common mechanisms [31, 35]. Of the four types of resistance (phenotypic and target resistance) is primarily identified by determining the knockdown time in minutes (KDT) and mortality rate to exposed insecticides (24 h post exposure) [35].

Insecticide resistance in malaria vector mosquito has already been documented in Tanzania, however the resistance levels has not reached level which can lead to operational failure [6, 36]. Despite the fact that, Tanzania records shows reduced susceptibility levels of malaria vectors against different insecticides in most areas other studies have shown marginal susceptibility in a number of sentinel sites [6, 34]. Low susceptibility to 0.75% permethrin have been reported in Arumeru, Lower Moshi, and Dar-es-salaam with post exposure of 92, 77 and 92% respectively [6, 34].

The aim of this study was to assess the insecticide use practice, knowledge, frequency of insecticide use and pattern, type of vector control tool and method of vector control on the phenotypic insecticides resistance and resistance ratio among malaria vectors *A. gambiae* s.l wild populations in Lower Moshi rice irrigation scheme to lambdacyhalothrin, permethrin, DDT and Bendiocarb.

## Methods

### Study area

This study was carried out in Lower Moshi (37°20′E3°21′S and 700 m altitude), an intensive rice-irrigation area, south of Mount Kilimanjaro in north-eastern Tanzania. Mosquitoes were collected from two hamlets (Mabogini and Rau Kati). These two hamlets were selected based on their agricultural practices differences. Most of the population in the area is engaged in agriculture and livestock production. Rice irrigation is the predominant activity although other crops such as beans, maize and green vegetables are grown for subsistence. Insecticides are used for control of insect pests in agriculture and livestock production as well as control of human disease vectors such as mosquitoes. Two rivers, Njoro and Rau provide water for irrigation. There are two growing seasons, the main one is from June to October and the second one involving sporadic cultivation of rice is from September to February.

### Sampling and sample selection technique

Semi-gravid adult *A. gambiae* s.l mosquitoes were collected between May and June 2013 from Mabogini and Rau Kati. The months of May and June are within the long rain season with high mosquito density. One central point was randomly selected from each village followed by random selection of the direction in which household interviews were conducted (simple random sampling technique). After household survey, the interviewer continued in the same direction interviewing every subsequent head of household or any adult above 18 years old available at the time of interview. In case of non-response (call backs were not implemented), the interviewer proceeded to the next household. Only one individual per household was interviewed. All households were visited in a multi-household dwelling.

### Data collection tools

Data collection for cross-sectional survey utilized structured questionnaire with both closed and open ended-questions. The questionnaire was designed to capture all variables for the study including demographic characteristics of the study population, name of insecticide (trade, common and generic), ingredient of insecticide, types of insecticides [lambdacyhalothrin, deltamethrin, permethrin and dichloro-diphenyl-trichloroethane (DDT)], type of vector control tools (integrated, biological, environmental management, chemicals), knowledge of insecticide use (manufacturer information, storage, dosage and concentration, safety precautions measures), frequency of insecticide application (daily, weekly, monthly), years of application, time of application (night/day), season of application, Insecticide application technique (spraying, smearing, dipping, impregnated in a targeted object, etc.), forms of insecticide (powder and concentrate, coils, sprays, wettable powder, insecticide chalks and jelly) and insecticide use (agriculture, veterinary or public health). Data collection tool for susceptibility test was a form capturing information relevant for the test to be carried as instructed in WHO guidelines [37]. The form captured information such as mosquito stage (adult/larvae) collection method (indoor/outdoor), types of breeding site (rice field, rainwater pool), mosquitoes information (age, species, date collected/and tested), insecticide information, storage condition, test results, knockdown time and mortality). Susceptibility tests B were carried out using WHO test kits for adults mosquitoes [37] with four insecticides Two pyrethroids [0.05% deltamethrin (DE 271, manufactured September, 2012 and expired September, 2013)] 0.75% permethrin [PE 192, manufactured September, 2012 and expired September, 2013)], carbamate [0.1% bendiocarb (BC 081, manufactured September, 2011 and expired September, 2014)] and organochlorides [4% DDT (DD 150, manufactured August, 2011 and expired August, 2016)]. Impregnated papers were obtained from the WHO Collaborating Centre in Penang, Malaysia. A minimum of 100 Anopheles mosquitoes (4 replicates of 25 mosquitoes each,) were collected for susceptibility test. The numbers of knockdown mosquitoes were recorded at interval of 10, 15, 20, 30, 40, 50, 60 min (1 h). Test was accompanied by control test where mosquitoes were exposed to paper treated with Silicone oil [Hangzhou Jessica Chemicals Co., Ltd (Pyrethroid control)] or risella oil [manufactured at Shell’s world-class Pearl GTL plant in Qatar (DDT control)] for 1 h. Bioassays were also carried out on the *A. gambiae* s.l Kisumu susceptible strain (KMS strain). After exposure, mosquitoes were kept in paper cups and supplied with a 10% sugar solution at 25–27 °C temperature, light regime of 12L:12D; relative humidity of 77 ± 2% and the mortality was recorded after 24 h.

### Mosquitoes sampling

A minimum of five houses were sampled randomly in the two hamlets every daily. Mosquitoes were collected using mechanical aspirator in cowshed [38]. Mosquitoes were placed in a paper cup covered with netting material and provided with 10% sucrose solution. They were placed in a cooler box and transported to the testing laboratory. Blood fed mosquitoes were left for 24 h in insectary to digest the blood meal to semi-gravid. Insectary light condition was light:dark (12L:12D) and relative humidity of 78 ± 2%. Mosquitoes were then used for insecticides susceptibility testing [31]. Laboratory susceptible colony was tested for insecticide resistance ratio calculation purposes.

### Insecticide susceptibility tests

To minimize the influence of a blood meal on exposures fully fed mosquitoes were left overnight to digest the blood meal before exposure to insecticides. Only female *A. gambiae* s.l. were used for the susceptibility tests according to WHO criteria [39].

### Morphological identification

Adult female *A. gambiae* s.l mosquitoes were identified after susceptibility testing. Morphological identification was done using a key that was developed by Gillies and Coetzee [32].

### Data management and analysis

Data were double entered and compared for consistency before analysis. The data were coded before entering them into Statistical Package for Social Scientists (SPSS) Software for analysis. Descriptive statistics (frequencies and percentages) were calculated to give characteristics of study variables. Cross tabulation was performed to determine relationships between choice of vector control methods/approach and other determinants (demographic characteristics, knowledge of insecticide use and practice, economic status, assets ownership and economy diversification) (Additional file 1). The P value was extracted and used to interpret the significance of the statistical test. Differences between groups compared were considered statistically significant when *P* < 0.05.

### Household sample size estimation

This sample size is estimated at 95% confidence level, 5% margin of error, and a proportion of 50% for unknown proportion of household knowledgeable on appropriate use of insecticide. A sample size formula is as shown below:

$$\text{N = }\frac{{{\text{Z}}^{2} {\text{P}}\,\left( {100 - {\text{P}}} \right)}}{{{\text{E}}^{2} }}$$

where N = Sample size, P = 50% of household knowledgeable on appropriate use of insecticide (assumption, proportion unknown), E = Margin of Error = 5%, Z = Level of Confidence, Z = 1.96 for 95% Confidence Interval. N = 1.96^2^ * 50(100 − 50), N = 384.

The 15% was added for non-responses, drop outs or missing data, the sample size taken was (0.15 * 384) + 384 = 441.6. The calculated sample size was rounded off to 450 participants.

### Data analysis

Probit analysis was used for analysis of mosquitoes susceptibility status to different insecticides [40]. In analysis, number of mosquitoes knocked down was considered as response frequency. Total number of mosquitoes used per test was considered as total number observed, Insecticides were considered as covariates and time was considered as a factor. Natural response was calculated from the data. In calculating the 24 h mortality post exposure, descriptive statistics was used in which exploration of the data was conducted by overall location, by type of insecticides and by both site and insecticides. Mortality was considered as dependent variable while site and insecticide was considered as factors. The fifty percent knockdown time (KDT_50_) recorded from field-collected mosquitoes from Lower Moshi was compared with that of the *A. gambiae* Kisumu reference susceptible strain by estimates of KDT_50_ and resistance ratio (RR). Abbott’s formula was not used to correct the observed mortality in adult susceptibility tests because there were no mortality in control group [41]. The World Health Organization standard criteria were used to evaluate the insecticides resistance/susceptibility status of the tested mosquito populations (a mortality in the range 98–100% indicates susceptibility); a mortality of <98% is suggestive of the existence of resistance and further investigation is needed; if the observed mortality (corrected if necessary) is between 90 and 97%, the presence of resistant genes in the vector population must be confirmed, and if mortality is <90%, confirms of the existence of resistant genes in the test population [37].

### Conceptual framework

**Fig. 1:**
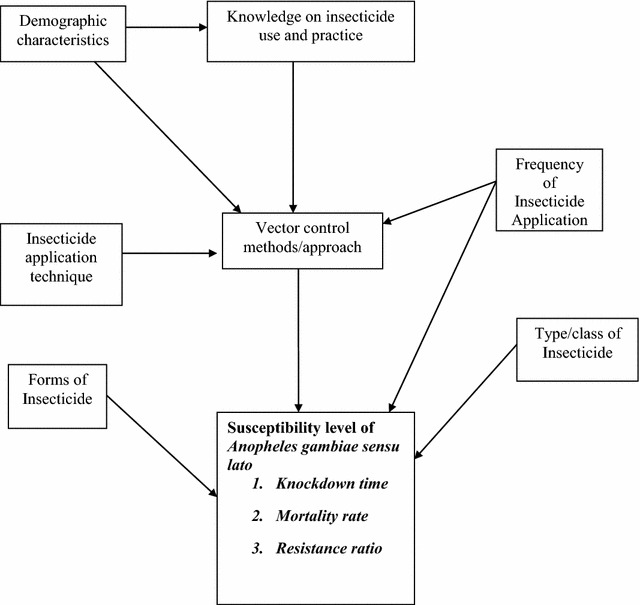
Conceptual framework which was used during the study planning

Many factors may contribute to susceptibility level of *A. gambiae* s.l. This includes vector control methods, types/class of vector control tool and knowledge of insecticides use and practice. However, on the other side vector control tool itself may be affected by demographic characteristics of respondents, knowledge of vector control and practice, insecticide application technique and frequency of insecticide application. The result of interrelation of all the factors may affect the susceptibility level as summarized by Fig. 1.

## Results

### Demographic characteristics of respondents

A total of 448 respondents participated in the study of which 39.7% (n = 178) were males and 60.3% (n = 270) were females. The mean age of respondents was 43.78 ± 13.491 and most of them were within the age group of 46–55 (41%, n = 181). The average number of people in a household was 4.75 ± 1.9 and majority of members have primary education (69.9%, n = 313). Most of the respondents were married (75.7%, n = 333) and many households found to have 0–2 children aged below 5 years (96.7%, n = 433). Total number of people in a household ranged between 4–6 (61.2%, n = 274) and the majority of the respondents reported farming as their main source of income (Table 1).

**Table 1:** Demographic characteristics of respondents (N = 448)

Characteristics	Frequency (n)	Percentage (%)
Sex		
Male	178	39.7
Female	270	60.3
Age group		
18–25	127	28.8
26–35	95	21.5
36–45	133	30.2
46–55	181	41.0
Education level		
Primary	313	69.9
Secondary	100	22.3
Others	35	7.8
No of people in the household		
1–3	117	26.1
4–6	274	61.2
5+	57	12.7
No of under-fives in the households		
0–2	433	96.7
3+	15	3.3
Marital status		
Single	51	11.6
Married	333	75.7
Others	56	12.7
^a^Major sources of income		
Farming	326	72.8
Livestock keeping	97	21.7
Fishing	10	2.2
Business	95	21.2
Employed	19	4.2
Others	35	7.8

This table was developed using descriptive and one way analysis of variance (ANOVA) statistics output

^a^Multiple response option

### Insecticide usage pattern

Majority (80.8%, n = 320) of respondents reported to have applied insecticides in the past 5 years mainly against crop pests (77.3%, n = 307). They also used insecticide for both veterinary (killing insects, 30%, n = 119), (nuisance control, 30.2%, n = 120)) and for household purposes (malaria vector control, n = 202, 30.2%). Generally reported trend of insecticides use increased for farming purposes (46.7%, n = 154) in the past 5 years while decreased for public health uses (69.3%, n = 158). The most commonly used pesticides were dursban (49.6%, n = 148) for farming, cybadip (71.1%, n = 83) for veterinary and Icon/Ngao (49.1%, n = 107) for public health pests. Through reading the label and material data sheet of the container/packaging, it was found that active ingredients contained in the insecticide were chloropyrifos (49.7%, n = 148) for farming, cypermethrin (77.9%, n = 81) for veterinary and lambdacyhalothrin (58.6%, n = 116) for public health purposes. It was further detected that major types of pesticides used were organophosphate for farming purposes (55.4%, n = 165) and pyrethroid for both veterinary and public health purposes (89.42%, n = 93; and 89.1%, n = 179) respectively (Table 2).

**Table 2:** Surveyed community insecticide use response pattern for farming, veterinary and domestic pests

S/no.	Characteristics	Activity		
		Farming, n (%)	Veterinary, n (%)	Domestic, n (%)
1	Proportion of insecticide use	320 (80.8%)	150 (37.9%)	238 (59.9%)
2	Main purpose			
	Insect killing	307 (77.3%)	119 (30%)	99 (25.0%)
	Repellant	44 (11.1%)	24 (6%)	21 (3.5%)
	Nuisance control	0	120 (30.2%)	
	Malaria mosquito control	0	0	202 (50.9%)
	Others	14 (35.5%)	14 (3.5%)	0
3	i. ITNs	NA	NA	371 (93.9%)
	ii. LLINs	NA	NA	361 (93.3%)
4	Trend of insecticide use in the past 5 years			
	Increasing	154 (46.7%)	45 (28.8%)	30 (13.2%)
	Decreasing	87 (26.450	81 (51.9)	158 (69.3%)
	Constant	89 (27.0%)	30 (19.2%)	40 (17.5%)
5	Type of insecticide ingredients			
	Chloropyrifos	148 (49.7%)	NA	NA
	Chlorpyrifos and cypermethrin	81 (27.2%)	NA	NA
	Lambdacyhalothrin	12 (4.0%)	NA	116 (58.0%)
	Endosulfan	27 (9.1%)	NA	NA
	Profenofos	20 (6.7%)	NA	NA
	Carbarly	0	9 (8.7%)	22 (11.0%)
	Cypermethrin	0	81 (77.9%)	NA
	Cypermethrin and tetramethrin	0	5 (4.8%)	52 (26.0%)
	Phenothrin and impothrin	NA	NA	10 (5.0%)
	Others (primiriphos-methyl, DDT, and dimethonate)	10 (3.4%)	9 (8.7%)	0
	Others	0	7 (4.2%)	11 (4.8%)
6	Class of pesticide frequently used			
	Pyrethroids	20 (6.7%)	93 (89.4%)	179 (89.1%)
	Organophosphates	165 (55.4%)	1 (1%)	0
	Organochlorides	31 (10.4%)	1 (15%)	0
	Carbamates	0	9 (8.7%)	22 (10.9%)
	Organochlorides and pyrethroids	82 (27.5%)	0	0

This table was developed using descriptive statistics and one way analysis of variance (ANOVA) output

*NA* not applicable

### Vectors control tools

Overall, most of respondents reported that insecticides (89.5%, n = 401) and environmental management (89.2%, n = 355) methods were used for vector control. However, very few respondents reported to use other vector control types (Table 3). Further analysis was done by producing composite variables for integrated methods used; combination of three or more methods (integrated) and non-integrated methods (2 or only 1 method). It was found that majority of the respondents used non-integrated method for vector control (75.9%, n = 302) compared to integrated ones (24.1%, n = 96).

**Table 3:** Vector control method options by respondents

Tool	Frequency (n)	Percentage (%)
1. Use of insecticide	401	89.5
2. Environmental management	355	89.20
3. Biological control (use of fish and fungus)	21	5.30
4. Other chemicals	94	23.60
5. Integrated method	17	4.30
6. Others (undefined)	7	1.80

This table was developed using descriptive and one way analysis of variance (ANOVA) output

### Knowledge of insecticide use and practice

Majority of respondents (85.10%, n = 330) agreed that, they are aware of where to get information on insecticide. The most common source of information cited was from insecticides dealers and distributers (67.20%, n = 262) and lastly by reading from insecticides material data sheet (48.30%, n = 189) (Table 4). Many respondents also reported to have knowledge on use of insecticides (91.20%, n = 330) of which looking on expiry date was frequently considered and also, reading package labels (45.3%, n = 178) (Table 4). Total knowledge was determined by recoding and combining variables for knowing source of information (know where to get information, extension officer and veterinary officer, material data sheet, container label) and important information considered (expiry date, certification log, container label, language on the label and know important information to consider before using or buying insecticide). It was found that almost half of respondents have high level of knowledge on insecticide use and practice (51.8%, n = 184) and the rest have low level of knowledge.

**Table 4:** Proportions of respondents’ knowledge on insecticide use and practice

No.	Indicator	Frequency (n)	Percentage (%)
1	Knowledge of where to get information of pesticide use	330	85.10
2	Source of Information		
	Veterinary and Public Health Officers	262	67.20
	Material data sheet	189	48.30
	Container/package label	131	33.60
	Others(TV, Radio, Friends, Seminar)	48	12.40
3	Important information to consider		
	Know important information	330	91.20
	Expiry date	307	78.10
	Container label	178	45.30
	Certification logo	148	37.70
	Language on the label	55	13.70

Data analysis output in this table was performed by descriptive statistics analysis

### The knock down time for wild *Anopheles gambiae* s.l. in Mabogini

A total of 4200 wild *Anopheles* s.l. adult mosquitoes were collected from May to June 2013 in the two hamlets (Mabogini and Rau Kati). In Mabogini, the least KDT_50_ was recorded for Bendiocarb and the highest for DDT. The KDT_95_ in Rau Kati was low for Bendiocarb but high for DDT (Table 5). Overall knockdown time was high in DDT, moderate in Pyrethroids (permethrin and deltamethrin) and lower in bendiocarb (Table 5).

**Table 5:** Mean knockdown time for wild *Anopheles gambiae* s.l

	Mean KDT50	KDT50 (95% CI)		Mean KDT95	KDT95 (95% CI)	
		Lower	Upper		Lower	Upper
Mabogini						
Permethrin	49.94	45.06	54.91	73.73	67.76	81.47
Deltamethrin	39.57	34.5	44.74	63.36	57.22	71.28
Bendiocarb	28.95	24.93	32.93	52.74	47.58	59.54
DDT	57.09	49.33	64.96	80.89	72.6	90.95
Rau Kati						
Permethrin	53.06	50.51	55.65	72.75	69.67	76.2
Deltamethrin	36.03	34.02	38.06	55.73	53.13	58.67
Bendiocarb	29.78	27.57	32	49.47	46.72	52.56
DDT	52.696	50.704	54.709	72.39	69.797	75.328

This table was developed using logistic regression analysis output

### Resistance ratio of wild *Anopheles gambiae* s.l. against laboratory susceptible colony

It was found that the resistance ratio of wild *A. gambiae* to laboratory colony based on KDT_50_ for bendiocarb, deltamethrin and permethrin is twice as that of Kisumu susceptible strain but for DDT it was almost the same as that of Kisumu susceptible strain (Table 6). The mortality ratio was highest for bendiocarb with 1.00 (Table 7).

**Table 6:** Resistance ratio of wild *Anopheles gambiae* s.l against susceptible laboratory strain for different insecticides

Insecticides	Wild population	Susceptible strain	Resistance ratio	P value
	KDT50 (95% CI)	KDT50 (95% CI)		
Permethrin	51.62 (49.18–54.10)	18.40 (15.90–20.87)	2.81	<0.001
Deltamethrin	37.08 (34.94–39.25)	15.16 (12.60–17.62)	2.45	<0.001
Bendiocarb	29.44 (27.36–31.54)	13.65 (11.01–16.15)	2.16	<0.001
DDT	53.49 (51.16–55.84)	57.45 (53.73–60.91)	0.93	0.081

This table was developed using logistic regression analysis output

**Table 7:** Mortality ratio of wild *Anopheles gambiae* s.l against susceptible laboratory colony based on 24 h mean mortality

Insecticides	24 h wild mortality	24 h laboratory colony mortality	Resistance ratio
Permethrin	89.68	100	0.90
Deltamethrin	69.96	100	0.70
Bendiocarb	100	100	1.00
DDT	99.23	100	0.99

This table was made with logistic regression statistics output

### Mean mortality of wild *Anopheles gambiae* s.l in 24 h

The study found that *A. gambiae* s.l was highly susceptible to bendiocarb and DDT (mortality rate of 100 and 99.2% respectively), increased tolerance to permethrin (mortality rate = 89.68%) and resistant to deltamethrin (mortality rate = 69.96%) (Table 8).

**Table 8:** Mean mortality after 24 h and knockdown time for wild *Anopheles gambiae* s.l

Insecticides (wild)	Number exposed (N)	Number of experiments	KDT_50_	95% CI		Mean mortality(%) after 24 h
				Lower	Upper	
Permethrin	680	4	51.62	49.18	54.10	89.68
Deltamethrin	680	4	37.08	34.94	39.25	69.96
Bendiocarb	680	4	29.44	27.36	31.54	100
DDT	680	4	53.49	51.16	55.84	99.23

This table was developed using logistic regression statistics analysis output

## Discussion

The present study investigated the insecticide usage pattern and phenotypic susceptibility of *A. gambiae* sensu lato to commonly used insecticides in Lower Moshi, northeastern Tanzania. Farming was reported to be the main income activity in the area and demographic characteristics were similar to other peri-urban areas of Tanzania as reported in the 2012 National Census Survey [42].

The proportion of pesticides used for farming in developing countries has been shown to be slightly higher compared to developed countries like Thailand whereby almost half of proportion of small scale farmers used insecticide [43]. The main reason why farmers use high amount of insecticides is to increase their yield through protecting crops against pests. Increased application of insecticide for farming purposes particularly for protecting crops against pests poses critical challenges as it may accelerate widespread of resistance strains of insects vector especially malaria vector in areas where agriculture is the main activity [35]. Even though linking of increases in insecticide resistance to farming has been previously reported, studies shows that resistance may differ in a short period of time, place and even at short distances [36, 44]. For example, in Kenya Mwea irrigation scheme where a lot of insecticides are used in rice production, *A. arabiensis* were found to be highly susceptible (with mortality of 94%) to all of insecticides recommended for malaria vector control [45]. This means that that there are no resistant genes in this population of malaria vectors. Monitoring the development of insecticide resistance in areas were insecticide based tools such as LLINs and IRS are being used should be reinforced to avoid compromising vector control interventions [26]. This study found that a majority of respondents reported usage of insecticides for malaria vector control in 93.3% of household. However, LLINs coverage findings differ from other Tanzania demographic and health survey in which the national coverage mean was about fifty percent (50%) while in this study was found to be 93.3% [45]. The difference can be justified by the reasons that at the time of Tanzania demographic and health survey (TDHS), LLINs were not yet distributed in all regions of mainland Tanzania. Despite this variation in coverage it can be concluded that the study area has exceeded the minimum target of millennium development goal of 80% coverage of LLINs at household level. The government of Tanzania has taken extra efforts in distribution and scaling up of LLINs for wide coverage and usage [10, 11]. It must be taken into cautions that high coverage and usage of LLINs has been associated to increased insecticide resistance of *A. gambiae* as in the case of Senegal [26]. High coverage of LLINs increases exposure of vectors to insecticides which causes them to be tolerant and spread the gene in wild populations of malaria vectors where the genes are already present [46, 47]. The study of Kulkarni and others showed that, the *A. gambiae* s.l and *A. funestus* remained highly susceptible with mortality rates of 87–100% despite long-term insecticide-treated net use [45].

Increased trend of insecticide usage for farming purposes and decreased use for veterinary and public health purposes in the past 5 years was reported during this study. Similar observations were also reported by in a study for small scale vegetable farmers in north Tanzania [48] In this study chloropyrifos and dursban was the main active ingredients and brand name reported respectively. Another study conducted recently by Nkya and others, substantiated a relationship between agriculture and insecticide resistance in disease vectors mainly mosquitoes by showing that, the intensity of pesticides usage is correlated with high resistance rates among malaria vectors [36]. The class and active ingredients of mostly applied pesticides reported in this study is similar to that reported by small scale farmers in Tanzania [48] and Thailand [43] the only differences was the brand names, however, active ingredients were the same.

Data on vector control tool usage showed that, environmental management and use of insecticides were the most prevalent vector control methods. Other approaches including biological control were least reported. Despite that environmental management reduces breeding sites of vectors the fewer survivors can still develop resistance due to high use of insecticides. Moreover, the reported use of biological vector control approach is rarely applied as compared to other places with irrigated rice practices in Middle East [49, 50]. In other studies done in Ethiopia, 28% of people reported the use of bio-pesticides such as fungus to control vectors especially malaria vector as the biological method and alternative for management of insecticide resistance [51]. The concept of Integrated Vector Management (IVM) was developed as a result of lessons learnt from integrated pest management, which is used in the agricultural sector; IVM aims to optimize and rationalize the use of resources and tools for vector control [52]. In other countries such as Zambia, application of IVM for malaria control have shown significant results in malaria reduction compared to where IVM was not applied [53]. Moreover, one should note that IVM approach makes vectors to be more susceptible to insecticides and hence reduce resistance. This implies that, since majority of participants do not apply IVM, it is probably be one of the contributing factor to the observed increased resistance for some insecticides used.

Majority of the study population was found to have primary education with basic reading and writing ability. Lack of secondary and tertiary education may reduce their capacity to read and understanding instructions. A study done in Ethiopia found that, 44.5% of respondents get information on insecticides by reading container or package label [51]. Reported results in Ethiopia are slightly higher compared to the findings of this study of which 33.60% could understand information of labels. This implies that, even if respondents know where to get insecticides and where to get information but poor capacity of reading and translating information properly may cause someone to miss important information.

The analysis of phenotypic susceptibility is often recommended for detecting resistance within population when it is in earlier stage for policy makers and vector control tool options [3, 4, 34, 54]. This study observed that the median knockdown time of *A. gambiae* has increased as compared with other study conducted in the same place [34, 55]. Similar studies also observed that median knockdown time when compared with that of sentinel site such as Meru, Kyela and Muleba has been raised too [6, 32, 33]. This implies that, susceptibility of *A. gambiae* to insecticides such as permethrin, deltamethrin and DDT in term of median knockdown time has been increased thus indicating that resistance has started to develop. It was found that, the use of pyrethroids was high with least use of DDT within the study site. However, it must be taken into caution that irrespective of low application of DDT there is a possibility of cross resistance between pyrethroids and DDT as it was commonly reported in other studies [6, 35, 55, 56]. This study further found that carbamates were not much in use. The low usage of the bendiocarb in other studies has been found to associate with vectors susceptibility status in carbamates compared to other insecticides, therefore good option for future malaria vector control as suggested elsewhere [54]. However, in other countries evidence of bendiocarb resistance has been reported [57].

The wild population of *A. gambiae* s.l was found to be highly susceptible to bendiocarb (mortality rate of 100%) and DDT (mortality rate of 99.2%) but resistant to permethrin (mortality rate of 89.68%) and deltamethrin (mortality rate of 69.96%). Of these four insecticides tested in Lower Moshi rice irrigation scheme it was found that bendiocarb showed promising effectiveness towards malaria vector control. This has been proved by its 100% mortality rate and low median knockdown time. Moreover, it has been found that, high effectiveness of bendiocarb in this area is attributed by the fact that, carbamate is least applied insecticide in the form of carbaryl for veterinary use. However, we should note that despite its effectiveness, *A. gambiae* resistance to bendiocarb (mortality 33.3%) has been recorded in other places of Africa [57]. Hence its application should incorporate practices for maintaining insecticide effectiveness such as IVM approach. Also, the present study has found that DDT is still highly effective (mortality rate of 99.2%) and the previous study in the same site had similar results [55]. However, in some other places DDT resistance has been documented in some areas including the Sahelian region of Burkina Faso [58]. In this study it has been reported that, DDT is either used at low rate or not applied and this may be the reason why resistance has not yet developed in the area [55]. Even in other places of Africa, *A. gambiae* s.l were observed to be resistant to permethrin [58]. Similar scenario was observed in deltamethrin. These results are similar to the findings in Burkina Faso whereby *A. gambiae* s.l were found to be resistant in all places with the exception of Orodara site [58]. The findings of this study are contrary to that of previously study conducted in the same area as it was observed that *A. gambiae* s.l was susceptible to deltamethrin [6]. This can be associated with increased use of pyrethroids in the area especially those which share the mode of action with deltamethrin including lambdacyhalothrin which is mainly used for agricultural and public health purposes.

The increased resistance ratio in pyrethroids (permethrin and deltamethrin) as compared to the previous study in the same area [6]. Moreover, when compared to others site such as Dar-es-salaam and Kilombero, similar findings of increased resistance ratio for pyrethroids were observed. Interestingly DDT showed much less resistance ratio as compared to all four insecticides tested and showed to be even much lower as compared with other sentinel sites such as Ilala, Kilombero and Arumeru [6]. The increased in resistance ratio among pyrethroids could probably be due to the use of insecticides in agriculture as reported in this study and this matched with other observations of previous studies done in Africa that, intensive use of insecticides might end up in insecticide resistance [36, 54]. Even if pyrethroids have shown to have increased resistance but is still suggested to be insecticides of choice to control malaria vectors because of relatively low toxicity to humans, rapid knock-down effect, relative longevity (duration of 3–6 months when used for IRS).

## Conclusion

*Anopheles gambiae* s.l was highly susceptible to bendiocarb and, increased tolerance to permethrin and deltamethrin. The most effective insecticide for malaria vector control observed in the study site was bendiocarb. Educational level was found to be a hindering factor to best practices for insecticide use in this area.

## Additional file

**Additional file 1.** The questionnaire used for data collection from head of households.

## Abbreviations

DDT: dichloro-diphenyl-trichloroethane
IRS: indoor residual spray
ITNs: insecticide treated nets
IVM: integrated vector management
KDT_50_: knockdown time for 50% of exposed population
KDT_95_: knockdown time for 95% of exposed population
KMS: strain-Kisumu susceptible strain
LLINs: long lasting insecticidal nets
SPSS: Statistical Package for Social Scientists
TDHS: Tanzania Demographic and Health Survey
WHO: World Health Organisation
