# Supplementary material for: Insecticide use pattern and phenotypic susceptibility of Anopheles gambiae sensu lato to commonly used insecticides in Lower Moshi, northern Tanzania
Source: BMC Res Notes. 2017 Sep 6;10:443. doi: 10.1186/s13104-017-2793-4 (PMC5585946; doi:10.1186/s13104-017-2793-4)
Supplement: Supplementary file 1 — Additional file 1. The questionnaire used for data collection from head of households. [file 13104_2017_2793_MOESM1_ESM.doc]

## Appendix I: Questionnaire

| **SN** | | **QUESTIONNARE IDENTIFICATION NUMBER** | | | | | | |  |
| --- | --- | --- | --- | --- | --- | --- | --- | --- | --- |
|  |  | **LOCATION PROFILE** | | | | | | | |
|  |  | **CODE** | **QUESTIONS** | **RESPONSE** | | | **CODE** | **QUESTIONS** | **RESPONSE** |
| 1 | | HOCD | Household code/number |  | | | 6. VISIT | Visit # |  |
| 2 | | DST | Name of District |  | | | 7. WARD | Name of Ward |  |
| 3 | | VILLAGE | Name of Village |  | | | 8. VEO | Name of Village Executive Officer |  |
| 4 | | INTERV | Name of Interviewer |  | | | 9. TIME | Time of Interview |  |
| 5 | | DATE | Date of interview | d__m__y___ | | |  |  |  |
| **HOUSEHOLD CHARACTERISTICS** | | | | | | | | | |
| 10 | | TFAMILY | Total Number of people in household |  | | | 13. CHILD | Total children below five years of age |  |
| 11 | | AGE | Age of respondents |  | | | 14. OWNER | Who own this house? | 1□Yourself 2□Family house 3□Rented Free 4□Others |
| 12 | | GENDER | Gender of respondent | 1□male, 2□Female | | |  |  |  |
| 15 | | EDU | What is your highest level of education? | 1□Primary 2□Form IV 3□Form VI 4□Technical school 5□College/University 6□No Formal education 7□Others_________ | | | | | |
| 16 | | MARITAL | Marital status | 1□Single 2□Married 3□Diforced 4□Widowed 5□Others__________ | | | | | |
| 17 | | ECONOM | Household main source of income | 1□Crop cultivation 2□Livestock keeping 3□Fishing 4□Business 5□Employment 6□Others____________ | | | | | |
| 18 | | INCOME | Approximate monthly income of household per month in monetary value | 1□<50,000 2□50,000-100,000 3□100,000-300,000 4□300,000-500,000 5□>500,000 | | | | | |
| 19 | | ASSETS | What types of assets are owned by your household? | 1□Farm 2□Cattle 3□Bicycle 4□Motorcycle 5□Car 6□Radio 7□Television 8□ Others____ | | | | | |
| **PESTICIDE INFORMATION AND USE PATTERN** | | | | | | | | | |
| 20 | USE | | Have you ever used an insecticide for any purpose | | | 1□Yes 2□No  (If NO please find another individual in the household or proceed to the next household) | | | |
| 21 | PURPOSE | | If YES for what purpose? (Tick All applicable) | | | 1□ Agriculture 2□Verterinary 3□Domestic | | | |
| 22 | AGRI | | If use for agriculture, for what purpose? | | | 1□Insects killing 2□Others (specify)_________ | | | |
| 23 | VERT | | If use for Veterinary, then for what purpose? | | | 1□Nuisance control 2□Insect killing 3□Repellent 4□Others (specify)_________ | | | |
| 24 | DOMEST | | If use for Domestic, then for what purpose? | | | 1□ Malaria vector control 2□Killing other Insects 3□Repellent 4□Others (specify)_________ | | | |
| 25 | OTHERM | | What other methods of vector control have you ever applied before | | | 1□Environmental management 2□ Biological control 3□Other chemical 4□Intergrated 5□others (specify)______________ | | | |
| 26 | CONAME | | Name the insecticide that you frequently use (common/local name) | | | 1□For Agriculture________________________________  2□For Veterinary________________________________  3□For Domestic_________________________________ | | | |
| 27 | INGRENAMES | | *Interviewer request for the insecticide package/container and for each of them write the generic, trade name and active ingredient* | | | 1□For Agriculture________________________________  2□For Veterinary________________________________  3□For Domestic_________________________________ | | | |
| 28 | TYPE | | *Interviewer should classify the type of insecticide named above as whether is*  *1□pyrethroid 2□Organophosphate 3□Organochoride*  *4□Carbamates* | | | 1□For Agriculture________________________________  2□For Veterinary________________________________  3□For Domestic_________________________________ | | | |
| 29 | TREND | | *What is the trend of insecticide use at the house hold level in past five years.* | | | - For Agriculture (1□= Increased , 2□Decrease D 3□ Remain constant ) - For Veterinary (1□= Increased , 2□Decrease D 3□ Remain constant ) - For domestic (1□= Increased , 2□Decrease D 3□ Remain constant | | | |
| 30 | SOURCE | | Where do you get insecticide you have mentioned above? | | | - For Agriculture1□= Agro vet shop, 2□Normal shop 3□Support from NGO/extension officer 4□Unaouthirised dealers 5□other---------- - For Veterinary 1□= Agro vet shop, 2□Normal shop 3□Support from NGO/extension officer 4□Unaouthirised dealers 5□Others ______ - For Domestic 1□= Health facility/Pharmacy/ADO/HCW, 2□Normal shop 3□Agrovet 4□Unaouthirised dealers 5□Others ______ | | | |
| 31 | INFO | | Where do you commonly get information on how to use insecticide you have mentioned? | | | - For Agriculture1□= Trained by a specialist , 2□From use manual container 3□ Trained by colleague 4□Use previous experience 5□other-------- - For Verterinary1□= Trained by a specialist , 2□From use manual container 3□ Trained by colleague 4□Use previous experience 5□other-------- - For Domestic1□= Trained by a specialist , 2□From use manual container 3□ Trained by colleague 4□Use previous experience 5□other-------- | | | |
| 32 | FORM | | In which form of insecticide you always apply it | | | - For Agriculture1□=powder & concentrate, 2□coils 3□wettable powder 4□jelly 5□Others_______________ For Veterinary 1□=powder & concentrate, 2□coils 3□wettable powder 4□jelly 5□Others_______________ For Domestic 1□=powder & concentrate, 2□coils 3□wettable powder 4□jelly 5□Others_______________ | | | |
| 33 | TECHNIQUE | | What technique you always use during application of pesticide? | | - For Agriculture (1□=spraying, 2□smearing 3□Dropings - For Veterinary ((1□=spraying, 2□smearing 3□dipping - For Domestic (1□=spraying, 2□smearing 3□Dropings 4□impregnated 5□Others | | | | |
| 34 | FREQUENCY | | How many times you always apply your insecticide? | | - For Agriculture (1□=daily, 2□weekly 3□monthly 4□annually 5□Others_______________) - For Veterinary (1□=daily, 2□weekly 3□monthly 4□annually 5□Others_______________) - For Domestic (1□=daily, 2□weekly 3□monthly 4□annually 5□Others_______________) | | | | |
| 35 | TIME | | At what time of the day you normally apply your insecticides? | | | - For Agriculture (1□=day time, 2□night time 3□Others_______________) - For Veterinary (1□=day time, 2□night time 3□Others_______________) - For Domestic (1□=day time, 2□night time 3□Others_______________) | | | |
| 36 | SEASON | | In which season of the year you mostly use the insecticide you have mentioned? | | | - For Agriculture (1□=dry, 2□rainy 3□betwen dry and wet) - For Veterinary (1□=dry, 2□rainy 3□betwen dry and wet) - For Domestic ((1□=dry, 2□rainy 3□betwen dry and wet) | | | |
| 37 | REASON | | State why you mostly apply insecticide in the mentioned season above? | | | 1□Agriculture____________________________________  2□Verterinary____________________________________  3□Domestic_____________________________________ | | | |
| 38 | ITN | | Do you use insecticide treated nets? | | | 1□Yes 2□NO | | | |
| 39 | LITN | | Is it long lasting insecticidal treated net? | | 1□Yes 2□NO | | | | |
| 40 | BOT | | Have you ever used any botanical repellents? | | 1□Yes 2□NO | | | | |
| 4i | WHY | | If YES, why? | |  | | | | |
| **KNOWLDEGE OF INSECTICIDE USE AND PRACTICE** | | | | | | | | | |
| 42 | KNOWINFO | | Do you know where to get information on how to use insecticide? | | | 1□Yes 2□NO | | | |
| 43 | WHERE | | If yes where do you get it? (Tick ALL apply) | | | 1□ From experts (extension officer, veterinary officer, health expert, ect)  2□ From material Safety Data Sheet  3□From container/packaging label  3□Others(specify)_____________________________ | | | |
| 44 | LABEL | | Do you know standard requirements to consider when buying insecticide | | | 1□Yes 2□NO | | | |
| 45 | REQUIRE | | If YES what main issue to consider? (Tick ALL apply) | | | 1□Expiry date 2□container label 3□cerfticiation logo 4□Language on the label 5□Others______________________ | | | |
